# Genome-wide identification and characterization of the Brassinazole-resistant gene family and associated responses to osmotic stress in *Avena sativa*

**DOI:** 10.3389/fpls.2025.1616026

**Published:** 2025-08-14

**Authors:** Shirui Xu, Zihao Wei, Mingchuan Ma, Lijun Zhang, Zhang Liu, Longlong Liu

**Affiliations:** ^1^ Center for Agricultural Genetic Resources Research, Shanxi Agricultural University, Taiyuan, Shanxi, China; ^2^ Germplasm Enhancement on Loess Plateau, Ministry of Agriculture and Rural Affairs, Taiyuan, Shanxi, China; ^3^ Houji Lab of Shanxi Province, Taiyuan, Shanxi, China; ^4^ College of Agriculture, Shanxi Agricultural University, Jinzhong, Shanxi, China

**Keywords:** *Avena sativa*, BZR transcription factor, genome-wide analysis, osmotic stress, gene expression

## Abstract

**Background:**

The Brassinazole-resistant (BZR) family of transcription factors acts as key regulators in brassinosteroid (BR) signaling, influencing plant growth, development, biotic and abiotic stresses. However, systematic analysis of the *BZR* genes in oat has not been conducted. Moreover, little is known about their functions in osmotic stress, which is a major abiotic stress affecting oat production.

**Methods:**

In this study, we performed a genome-wide analysis of the *BZR* gene family in oat. Their chromosome locations, gene structures, phylogenetic relationships, conserved domains, promoter cis-elements, and gene duplication events were analyzed. Furthermore, the expression patterns of *BZR* genes under osmotic stress were characterized, and the subcellular localization of AsBZR12 was investigated in *Nicotiana benthamiana*.

**Results and discussion:**

In this study, we mapped 14 members of the *BZR* gene family across 12 oat chromosomes, and classified them into three groups based on phylogenetic analysis. The BZR proteins displayed group-specific patterns in their exon-intron structures and conserved motifs. Furthermore, cis-acting element analysis revealed that *AsBZR* genes are primarily involved in phytohormone responses and environmental stress adaptation. Examination of gene duplication revealed that segmental duplications drove the expansion of the *BZR* gene family in the oat genome, with evidence of strong purifying selection pressure during evolutionary development. The qRT-PCR analysis demonstrated varied expression patterns among *AsBZR* members. Specifically, *AsBZR12* was significantly upregulated in roots, stems, and leaves, with nuclear localization. In summary, our study provides a comprehensive analysis of the *AsBZR* genes and characterizes their expression patterns under osmotic stress conditions, thereby identifying potential candidate genes for future research. This research provides comprehensive insights into *BZR* gene structure and evolution, establishing a foundation for understanding their osmotic stress responses in oat.

## 1 Introduction

Brassinosteroids (BRs) are an indispensable class of plant steroidal hormones that play crucial roles in regulating plant growth and development, as well as in coping with biotic and abiotic stresses (Manoli et al., 2018; Feng et al., 2023). The Brassinazole-resistant (BZR) transcription factor family has been extensively studied for its critical role in modulating BR signaling pathways (Wang et al., 2024). ATP-binding cassette (ABC) transporter ABCB19 mediates the transport of BRs to the apoplast, where these molecules bind to the plasma membrane-localized receptor BRASSINOSTEROID INSENSITIVE1 (BRI1) and its coreceptor BRI1-ASSOCIATED RECEPTOR KINASE 1 (BAK1), thereby initiating the BR signaling pathway (Li et al., 2002; Nam and Li, 2002; Ying et al., 2024). This interaction activates key transcription factors such as BRASSINAZOLE RESISTANT1 (*BZR1*) and BRI1-EMS-SUPPRESSOR1 (*BES1*)/*BZR2*, which are central to the regulation of gene expression in response to BR signaling (Lee et al., 2024). Despite sharing 88% amino acid sequence similarity, and 97% consistency in the DNA binding domain, *BZR1* and *BES1*/*BZR2* exhibit distinct functions (Yin et al., 2002; Li et al., 2024). *BZR1* functions as a transcriptional repressor that targets the BR response element (CGTGT/CG sequence). *BZR1* regulates BR homeostasis and signaling through a dual-action mechanism: it activates the expression of growth-promoting genes by recruiting the BRAHMA-associated SWI/SNF chromatin-remodeling complex to open chromatin, while repressing BR-responsive genes by interacting with TOPLESS to recruit histone deacetylase 19 and form a transcriptional repression complex (He et al., 2005; Zhu T. et al., 2024). In contrast, *BES1*/*BZR2* binds to E box (CANNTG) sequences and acts as a negative regulator of the BR pathway (Yin et al., 2005).

In the context of modern agriculture, climate change has emerged as a pivotal determinant of crop productivity. Rising global temperatures and erratic precipitation patterns have increased the frequency of drought conditions, making drought one of the most significant abiotic stresses affecting plant growth, development, and yield (Singh and Laxmi, 2015; He Z. H. et al., 2024; Zhan et al., 2024). An increasing number of studies have demonstrated that members of the *BZR* gene family play significant roles in plants’ responses to drought stress. In *Setaria italica*, *SiBZR1* functions as a negative regulator of drought tolerance by directly targeting *SiPLT-L1*, whose overexpression enhances drought resistance (Zhao et al., 2021). Similarly, in *Zanthoxylum armatum* DC, *BZR* genes are upregulated under drought stress, and overexpression of *ZaBZR1* in transgenic *Nicotiana benthamiana* confers enhanced drought tolerance (Jin et al., 2024). Moreover, *TaBZR2* in wheat positively regulates drought tolerance by activating *TaPPR13*, which enhances drought tolerance through facilitating ABA-mediated stomatal movement (Cui et al., 2019; Hou et al., 2025). Additionally, the response of *BZR* gene family members to drought stress has been reported in several species, including *Glycine max*, *Cucumis sativus*, *Populus trichocarpa*, and *Solanum tuberosum* (Li et al., 2018; Luo et al., 2023; Li et al., 2024; Wang et al., 2024). However, the role of the *BZR* gene family in drought stress response in oat remains unclear.

Oat (*Avena sativa* L.) is an allohexaploid (AACCDD, 2*n* = 6*x* = 42) plant with a large and complex genome. As a highly nutritious cereal crop, it plays a significant role in both human food and animal feed (Peng et al., 2022). The prominence of oats in scientific research and industry has increased owing to their health-promoting attributes, such as the cholesterol-lowering effects of oat beta-glucan and their potential benefits in managing type-2 diabetes (Othman et al., 2011; Guo et al., 2023). Moreover, oats contain unique phytochemicals, including avenanthramides and steroidal saponins, which exhibit antioxidant, anti-inflammatory, and potential anticancer activities (Paudel et al., 2021). Oats demonstrate robust adaptability to diverse soil and climatic conditions, enabling their cultivation in areas with harsh environmental stressors that are unsuitable for other food crops (Chen et al., 2024). Predominantly grown in the arid regions of northern, northwestern, and southwestern China, oat yields are significantly limited by dry climatic conditions (Zhang et al., 2021). The availability of a high-quality oat reference genome has greatly enhanced our understanding of their evolutionary history and facilitated the identification of functional genes (Kamal et al., 2022; Peng et al., 2022).

Recent research has increasingly focused on various oat gene families, such as the glutathione reductase gene family, *WRKY* transcription factor family, catalase gene family, MYB transcription factor family, and aquaporin gene family (Liu et al., 2022; Sun et al., 2022; Ghorbel et al., 2023; Chen et al., 2024; Zhou et al., 2024). To date, the *BZR* gene family in oat has not been comprehensively identified or analyzed, and the functional studies of *AsBZR* genes in osmotic stress response remain uncharacterized. In this study, we conducted a genome-wide characterization of *BZR* genes in *A. sativa*, elucidating their chromosome locations, gene structures, phylogenetic relationships, conserved domains, promoter cis-elements, and gene duplication events. Additionally, we examined the expression patterns of *AsBZR* genes under osmotic stress, revealing that *AsBZR12* was significantly upregulated in roots, stems, and leaves. Subsequently, we examined the subcellular localization of AsBZR12 in *Nicotiana tabacum*. The primary objective of this study was to conduct a comprehensive analysis of the *AsBZR* genes and to identify potential osmotic stress-responsive candidate genes by examining their expression patterns under PEG-induced osmotic stress conditions. These findings lay a foundation for future functional studies on the *AsBZR* genes in osmotic stress response.

## 2 Materials and methods

### 2.1 Identification and characterization of *BZR* gene family members in *A. sativa*

To perform genome-wide analysis in *A. sativa*, genome and annotation data were retrieved from the GrainGenes database (https://wheat.pw.usda.gov/GG3/content/avena-sang-download) (Kamal et al., 2022). To identify all possible *AsBZR* genes in *A. sativa*, both BLASTP and Hidden Markov Model (HMM) searches were performed. Known BZR protein sequences, including 6 from *Arabidopsis thaliana* (https://www.arabidopsis.org/) (**Supplementary Table S1**) and 6 from *Oryza sativa* (https://rice.uga.edu/) (**Supplementary Table S1**), were used as query sequences for BLASTP analysis with an e-value threshold set to less than or equal to 1e-5 (Ullah et al., 2022). In addition, oat protein sequences were searched against HMM profiles of the BZR domain (PF05687). To validate the presence of BZR domains in the candidate *AsBZR* genes, we performed manual domain annotation using three independent databases: Pfam (https://pfam-legacy.xfam.org/), the Conserved Domain Database (CDD) of the National Center for Biotechnology Information (NCBI) (https://www.ncbi.nlm.nih.gov/Structure/cdd/wrpsb.cgi), and SMART (http://smart.embl-heidelberg.de/) (Letunic et al., 2021). This analysis confirmed that all candidate genes contained the characteristic BZR conserved domain. The physicochemical properties of the AsBZR protein were calculated using the Protein Parameter Calculator in TBtools (Chen et al., 2023). Furthermore, the subcellular localization of the AsBZR proteins was predicted using Cell-PLoc 2.0 (http://www.csbio.sjtu.edu.cn/bioinf/Cell-PLoc-2/) (Chou and Shen, 2008).

### 2.2 Conserved motifs and gene structure analysis of AsBZR

The conserved motifs in the AsBZR proteins were identified using the MEME online program (https://meme-suite.org/meme/tools/meme) with the maximum number of motifs set to 10. The gene structure of the *AsBZR* genes was analyzed using the Visualize Gene Structure program in TBtools software based on *A. sativa* annotation data. The conserved motifs and gene structure information were visualized and mapped using the Gene Structure View program in TBtools.

### 2.3 Cir-acting and chromosome localization analysis

To identify the cis-acting elements in the promoter region, we screened 2000 bp upstream sequences of the *AsBZR* genes using PlantCARE tool (https://bioinformatics.psb.ugent.be/webtools/plantcare/html/). Based on the *A. sativa* annotation data, the chromosomal locations of the *AsBZR* genes were determined using the Gene Location Visualization tool in TBtools.

### 2.4 Phylogenetic tree construction and collinearity analysis

To analyze the evolutionary relationships and origins of *BZR* transcription factors from *A. sativa*, *Triticum aestivum*, *O. sativa*, and *A. thaliana*, we aligned their full-length amino acid sequences using the ClustalW program. The phylogenetic tree was then constructed with 1000 bootstrap replicates using MEGA 7.0 software with the neighbor-joining method. The phylogenetic tree was edited and visualized using the Interactive Tree Of Life (iTOL) platform (https://itol.embl.de/). Gene duplication events within the *AsBZR* gene family and synteny analysis of orthologous BZR genes from *A. sativa*, *A. thaliana*, *T. aestivum*, and *O. sativa* were performed using the One Step MCScanX program in TBtools, with an e-value threshold of 1e-10. The non-synonymous substitution (*Ka*) and synonymous substitution (*Ks*) substitution rates for each duplicated gene pair were calculated using the Simple *Ka*/*Ks* Calculator in TBtools.

### 2.5 Plant materials and treatments

*A. sativa* cv. Pinyan No.8 (abbreviated as PY8), provided by the Center for Agricultural Genetic Resources Research, Shanxi Agricultural University, was used in this study. Oat seeds were surface-sterilized with 1.0% sodium hypochlorite for 20 min and then washed three times with sterile water. The seeds were germinated on filter paper in petri dishes for 4 days and then transplanted to a germination box containing modified Hoagland nutrient solution. The experiment was conducted in a greenhouse under a 16 h light/8 h darkness cycle with a light intensity of 250 μmol m^-2^ s^-1^. At the one-leaf stage, PY8 seedlings were treated with 15% polyethylene glycol 6000 (PEG 6000) solutions. The first leaves and roots of PY8 seedlings were sampled at five time points (6, 12, 24, 48, and 72 hours), with three biological replicates in both treated and untreated groups. Additionally, the stems were collected at the 24-hour time point, with three biological replicates for each group. Each sample was immediately frozen in liquid nitrogen and stored at −80°C for further analysis.

### 2.6 Quantitative reverse transcription PCR

Total RNA was extracted from each sample using the DP432 kit (Tiangen, China), and cDNA was synthesized using HiScript III 1st-Strand cDNA Synthesis Kit (Vazyme, China). qRT-PCR was performed on the QuantStudion 6 Flex Real-Time PCR System (Thermo Fisher Scientific, USA) using 2X RealStar Fast SYBR qPCR Mix (Genstar, China). The primers used to evaluate the transcript levels are listed in **Supplementary Table S2**. The *AsActin* gene was used as the endogenous control. Relative expression levels were determined using the (2^−ΔΔCt^) ± SEM method. Each sample was analyzed in three biological replicates.

### 2.7 Subcellular localization analysis

To determine the molecular characteristics of the AsBZR12 protein, the coding sequence of *AsBZR12* was cloned and fused into the pCAMBIA1302-GFP vector. The recombinants and pCAMBIA1302-GFP empty controls were subjected to *Agrobacterium* (GV3101)-mediated transient expression in *N. tabacum* leaves, together with the nucleus marker plasmid NLS-mCherry. The transformed *N. tabacum* leaves were observed using confocal laser-scanning microscopy (LSM800, Carl Zeiss, Germany). The subcellular localization of AsBZR12 protein was determined at three times.

## 3 Results

### 3.1 Identification of *BZR* gene family in *A. sativa*

According to BLASTP and HMM searches, followed by examination of conserved domains in Pfam, NCBI-CDD, and SMART, a total of 14 *BZR* genes were identified in *A. sativa*. The *AsBZR* gene family members were systematically assigned and named *AsBZR1* to *AsBZR14* based on their respective chromosomal locations (**Table 1**). The amino acid length of AsBZR proteins ranged from 322 to 685 residues, with isoelectric points (PI) varying from 5.36 to 9.45, averaging 7.89. The predicted molecular weight of BZR proteins ranged from 34.71 kDa to 75.99 kDa, with a mean of 47.42 kDa. Proteins with an instability index above 40 are considered unstable and prone to degradation (Ullah et al., 2022). As the instability index of all AsBZR proteins was above 40, these proteins are likely unstable in *A. sativa*. Additionally, the grand average of hydropathicity (GRAVY) values for all AsBZR proteins were negative, indicating their hydrophilic nature. Subcellular localization prediction showed that all *AsBZR* genes were located in the nucleus.

**Table 1:** The identified *BZR* genes in *A. sativa*.

Gene name	Gene ID	Chromosome location	Amino acid number	PI	Molecular weight (Kda)	Instability index	Aliphatic index	GRAVY	Predicted subcellular location	Group
*AsBZR1*	AVESA.00010b.r2.1AG0028220.1	1A: 399201668-399208297	655	5.97	73.36	43.61	73.73	-0.33	Nucleus	Group III
*AsBZR2*	AVESA.00010b.r2.2DG0354460.1	2D: 201686654-201687751	325	9.09	35.08	73.56	56.52	-0.70	Nucleus	Group II
*AsBZR3*	AVESA.00010b.r2.2DG0389390.1	2D: 61361913-61368581	657	6.16	73.66	42.19	72.77	-0.36	Nucleus	Group III
*AsBZR4*	AVESA.00010b.r2.3AG0419990.1	3A: 65179990-65182682	360	8.59	38.13	66.77	52.61	-0.56	Nucleus	Group I
*AsBZR5*	AVESA.00010b.r2.3CG0467490.1	3C: 74958306-74961024	358	8.76	38.03	65.02	51.82	-0.56	Nucleus	Group I
*AsBZR6*	AVESA.00010b.r2.3DG0521200.1	3D:21787311-21790498	359	8.76	38.05	66.46	52.76	-0.57	Nucleus	Group I
*AsBZR7*	AVESA.00010b.r2.4CG1285820.1	4C: 148864431-148867947	355	7.70	37.53	65.63	57.21	-0.64	Nucleus	Group I
*AsBZR8*	AVESA.00010b.r2.5AG0861680.1	5A: 474385867-474390093	685	5.36	75.99	47.84	74.66	-0.42	Nucleus	Group III
*AsBZR9*	AVESA.00010b.r2.6AG1053720.1	6A: 352484986-352486760	322	8.80	34.71	75.31	57.64	-0.65	Nucleus	Group II
*AsBZR10*	AVESA.00010b.r2.6CG1103130.1	6C: 253118452-253121880	332	9.45	35.24	66.21	50.09	-0.63	Nucleus	Group I
*AsBZR11*	AVESA.00010b.r2.6CG1118530.1	6C: 132176749-132183329	653	5.94	73.37	44.15	72.16	-0.37	Nucleus	Group III
*AsBZR12*	AVESA.00010b.r2.6DG1182890.1	6D: 29420230-29423788	340	9.45	36.11	69.86	51.71	-0.61	Nucleus	Group I
*AsBZR13*	AVESA.00010b.r2.7AG1248040.1	7A: 483645003-483648748	352	8.20	37.32	65.80	57.70	-0.63	Nucleus	Group I
*AsBZR14*	AVESA.00010b.r2.7DG1359760.1	7D: 386680028-386683583	352	8.20	37.32	65.80	57.70	-0.63	Nucleus	Group I

### 3.2 Chromosomal distribution of *AsBZR* genes

The 14 identified *AsBZR* genes were mapped to 12 of the 21 oat chromosomes (**Table 1**), revealing an uneven distribution across the genome. The *AsBZR* genes were found on chromosomes 1A, 2D, 3A, 3C, 3D, 4C, 5A, 6A, 6C, 6D, 7A, and 7D, with each chromosome containing one to two genes. Specifically, chromosomes 2D and 6C each harbored 2 *AsBZR* genes, while single genes were present on the remaining chromosomes. Notably, homoeologous group 6 contained the highest number of *AsBZR* genes, comprising *AsBZR9*, *AsBZR10*, *AsBZR11*, and *AsBZR1*2. The *AsBZR* genes demonstrated relatively uniform distribution among the A, C, and D sub-genomes, with 5 genes in both A and D sub-genomes and 4 genes in the C sub-genome.

### 3.3 Phylogenetic analysis of *AsBZR* genes

**Figure 1:**
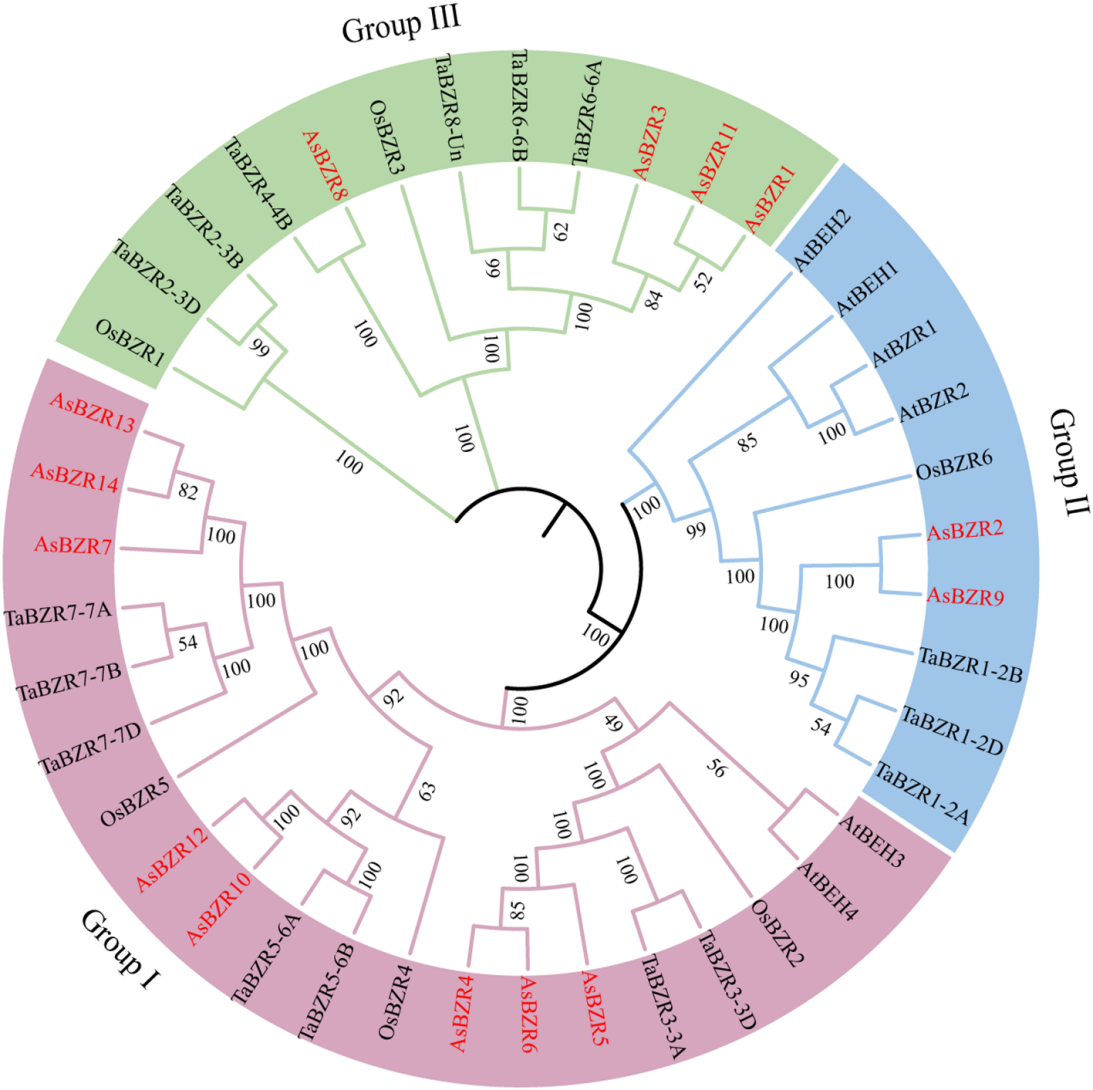
Phylogenetic relationship of BZR proteins in the *A. sativa*, *A. thaliana*, *O. sativa*, and *T. aestivum*. All AsBZR proteins are highlighted in red, and different colors represent different groups.

**Figure 2:**
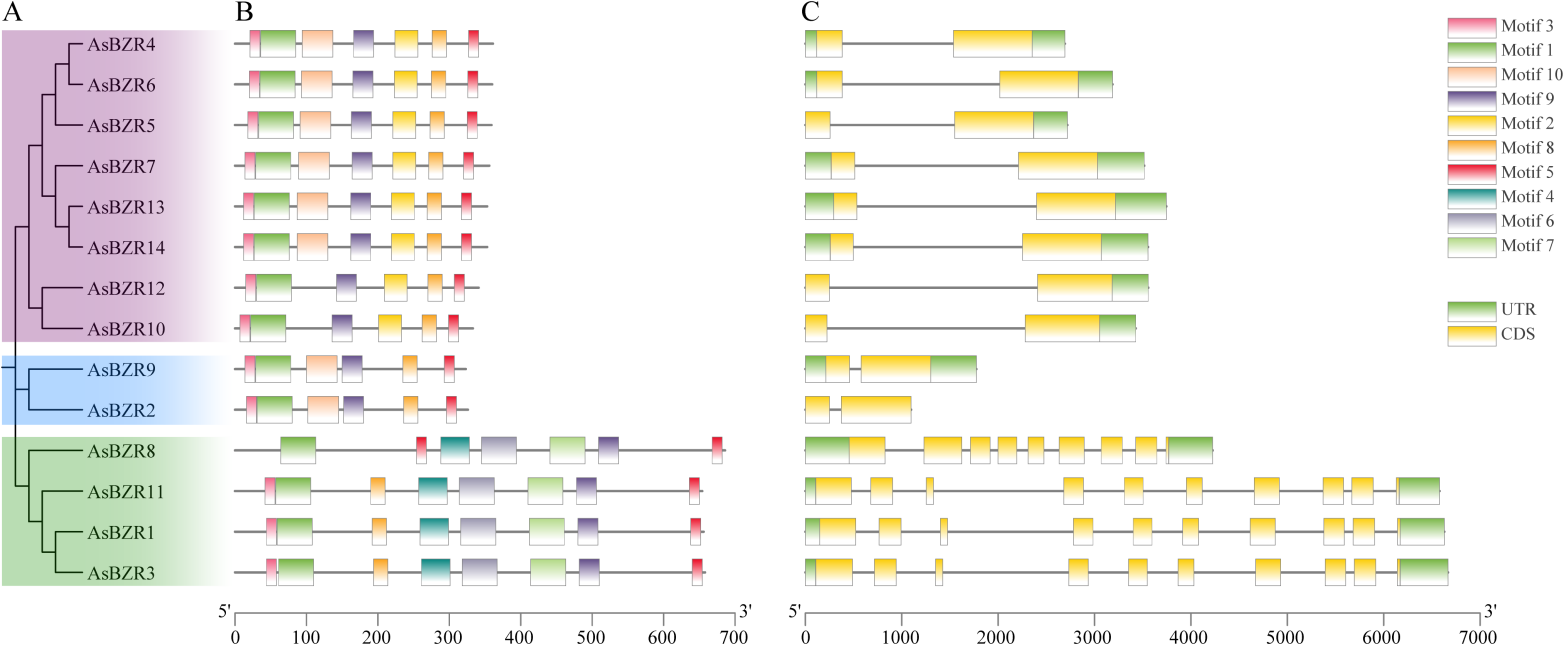
Phylogenetic tree, conserved motifs, and gene structure of *AsBZR* genes. **(A)** Phylogenetic tree constructed using the AsBZR protein sequences. **(B)** Distribution of conserved motifs for *AsBZR* proteins. The different motifs are shown in different color boxes. **(C)** Gene structure of *AsBZR* genes. Green boxes indicate UTR regions, yellow boxes indicate exons, grey lines indicate introns.

To elucidate the evolutionary relationships of *BZR* genes across species, the phylogenetic tree was constructed incorporating 14 AsBZR, 16 TaBZR, 6 OsBZR, and 6 AtBZR proteins (Ullah et al., 2022; Yin et al., 2005). The phylogenetic analysis revealed three distinct groups among the 14 AsBZR proteins. Among the three groups, Group I was the largest, with eight members: AsBZR4, AsBZR5, AsBZR6, AsBZR7, AsBZR10, AsBZR12, AsBZR13, and AsBZR14. Group II contained two members (AsBZR2 and AsBZR9), and Group III included four members (AsBZR1, AsBZR3, AsBZR8, and AsBZR11) (**Figures 1**, **2A**).

### 3.4 Gene structure and motif analysis of *AsBZR* genes

Analysis of sequence conservation in *AsBZR* genes revealed distinct patterns in their conserved motifs (**Figure 2B**; **Supplementary Figure S1**). Motifs 1, 5, 8, and 9 were widely distributed across all *AsBZR* domains, indicating their high conservation among the genes. Members within the same subgroup displayed similar patterns in both type and number of conserved motifs, suggesting functional similarities. The key distinction between Group I and Group II members was the presence of motif 2 exclusively in Group I, while motifs 4, 6, and 7 were characteristic of Group III.

To investigate the evolution of the *BZR* gene family in oat, we analyzed the exon-intron structures of 14 *AsBZR* genes. The analysis revealed that all *AsBZR* genes contained between two to ten exons (**Figure 2C**). *AsBZR* genes within the same group exhibited similar structural patterns. While *AsBZR* genes in both Group I and Group II contained one intron and two exons, Group I displayed longer intron lengths compared to Group II. Group III members contained nine to ten exons (*AsBZR8* with nine exons, *AsBZR1*, *AsBZR11*, and *AsBZR3* with ten exons). The phylogenetic analyses, conserved motif patterns, and similar gene architectures among *BZR* gene family members within clusters provide substantial evidence supporting the accuracy of the taxonomic classification.

### 3.5 Cis−acting elements analysis

**Figure 3:**
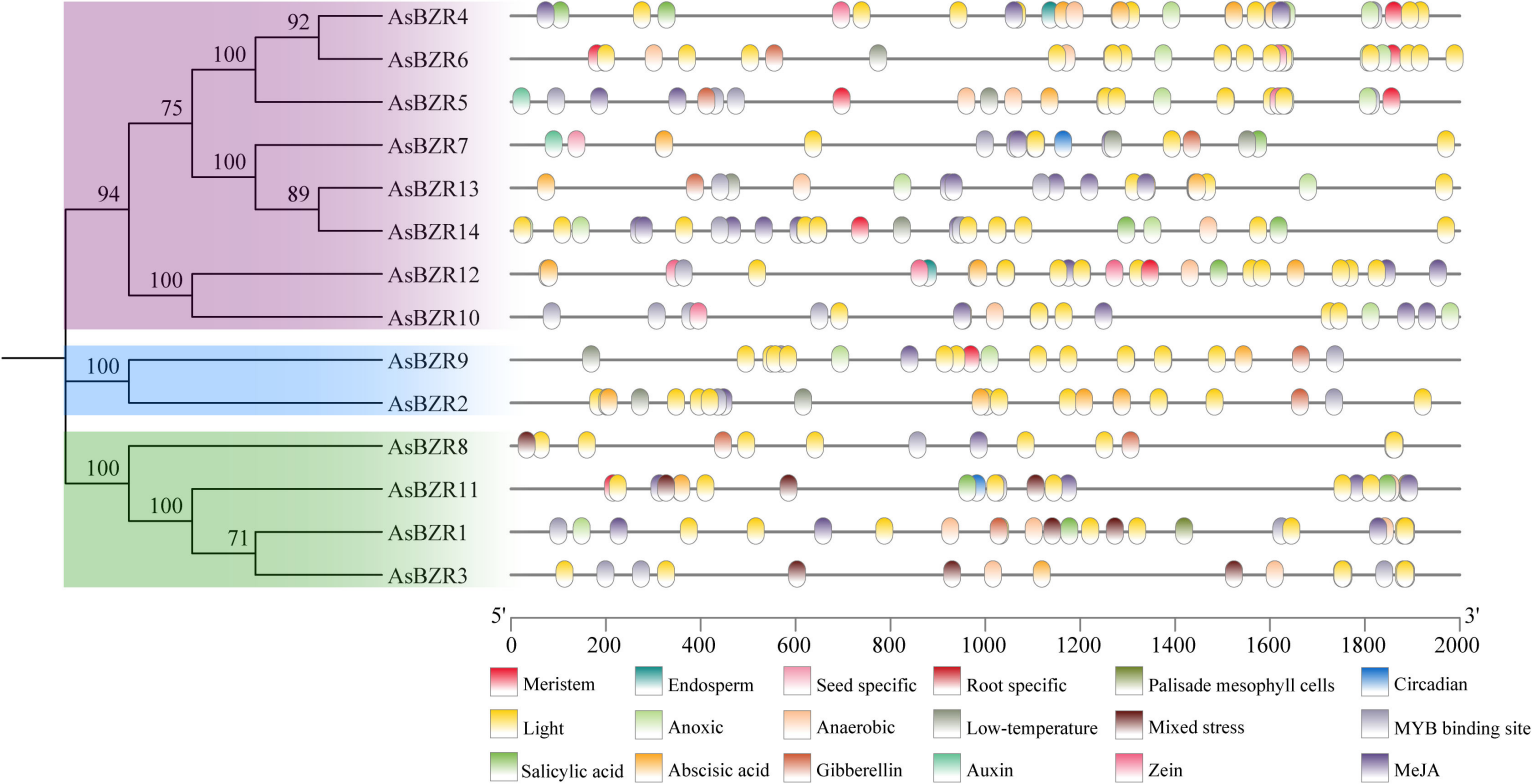
Cis-acting elements in the promoters of *AsBZR* genes. Different colors represent different elements.

To better understand the potential regulatory mechanism of the *BZR* gene family in oat, we examined the regulatory elements located 2000 bp upstream of the promoters for all 14 *AsBZR* genes (**Figure 3**; **Supplementary Figure S2**). The results showed that the promoters of *AsBZR* genes contain numerous phytohormones-responsive cis-elements, including those responsive to salicylic acid, abscisic acid, zein metabolism, methyl jasmonate (MeJA), gibberellin, and auxin. Additionally, cis-elements associated with environmental stress signal responsiveness were identified, including light, low-temperature, circadian, anoxic, anaerobic, and mixed stress (defense and stress responsiveness) elements. Several *AsBZR* genes contained tissue-specific expression elements for meristem, seed, root, and palisade mesophyll. Moreover, cis-elements associated with transcription factors, such as *MYB*, were found to bind to *BZR* gene promoters. The findings indicate that *AsBZR* family members likely participate in diverse stress and hormone signaling pathways, suggesting significant roles in stress responses and development.

### 3.6 Gene duplication and collinearity analysis of *BZR* gene family

**Figure 4:**
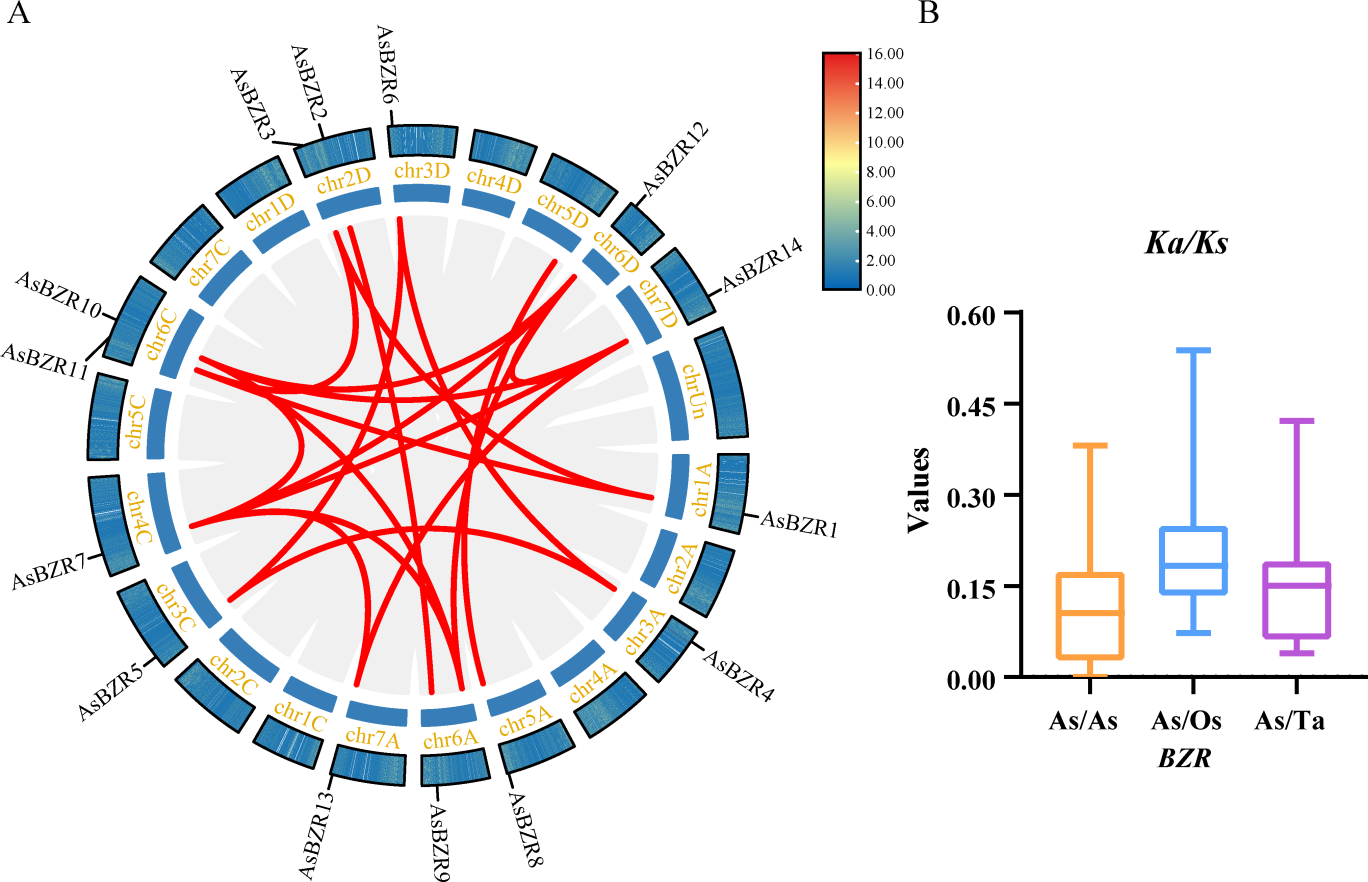
Duplication events and evolutionary rates of the *AsBZR* genes. **(A)** Duplication event in *A. sativa*. The gray lines indicate collinearity and the red lines highlight the duplication events of *AsBZR* genes. **(B)** Ka/Ks ratios of *AsBZR* gene pairs with *TaBZR* and *OsBZR*.

To elucidate the relationship between the expansion of the *BZR* gene family and the polyploidization process in oat, we performed a genome-wide analysis of duplication events involving *AsBZR* genes (**Figure 4A**). The analysis revealed 67,910 regions of collinearity in the oat genome, including those on the Un chromosomes. The study identified 19 segmental duplication events among 14 *AsBZR* genes, with some *AsBZR* genes having multiple repeats. No tandem duplication pairs were detected (**Supplementary Table S3**). Among these, 15 gene pairs were comprised of *AsBZR* genes at both ends, while the remaining pairs contained an *AsBZR* gene at one end and a non-*AsBZR* gene at the other. This indicates that the *BZR* gene family expansion in the oat genome occurred exclusively through segmental duplication, with no evidence of tandem duplication events.

To further elucidate the evolutionary dynamics and selective pressures affecting the duplicated *AsBZR* genes, we calculated the ratios of *Ka* and *Ks* substitutions for the 15 duplicated *AsBZR* gene pairs (**Supplementary Table S4**). The value of *Ka*/*Ks* > 1 indicates positive selection, = 1 indicates neutral selection, and < 1 suggests purifying selection (Liu et al., 2022). The results indicated that *Ka*/*Ks* values for the 15 gene pairs ranged from 0.00 and 0.38. Since these *Ka*/*Ks* values were all less than 1 (**Figure 4B**), this suggests that the oat *BZR* gene family has experienced strong purifying selection pressure during evolution. Additionally, we performed *Ka/Ks* ratio analyses of the *BZR* genes in oat compared with their orthologs in *O. sativa*, and *T. aestivum* (**Figure 4B**; **Supplementary Table S8**). The results showed that the *Ka/Ks* values were all less than 1, indicating that purifying selection has been a significant factor in the evolution of the *BZR* genes.

**Figure 5:**
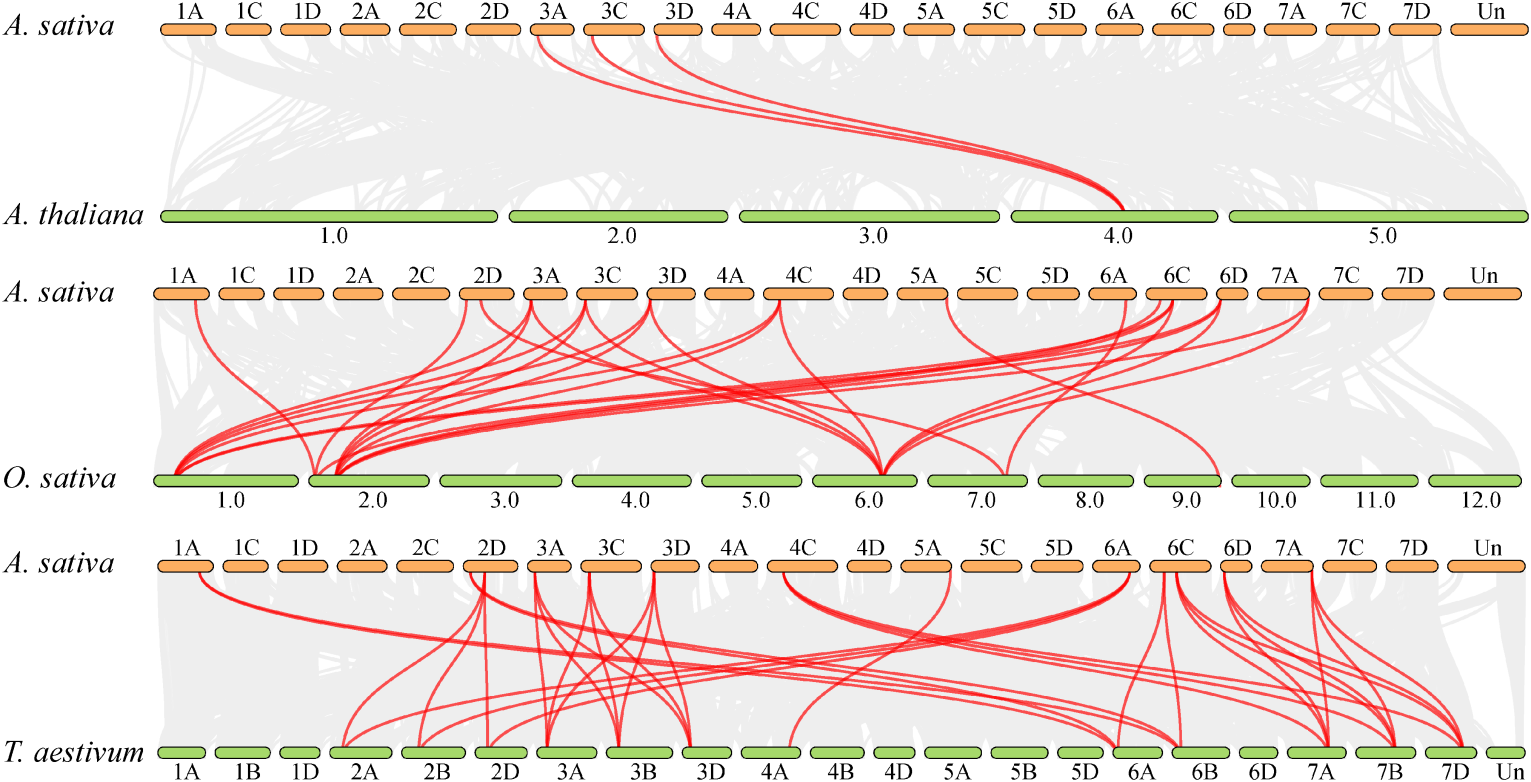
Synteny analysis of *BZR* genes between *A. sativa*, *A. thaliana*, *O. sativa*, and *T. aestivum*. The gray lines show colinear blocks within *A. sativa* with other plant genomes, while the red lines highlight the synteny *BZR* gene pairs. Chromosome numbers are labelled above or below each chromosome.

To investigate the evolutionary mechanisms and orthologous relationships of the oat *BZR* gene family, comparative synteny maps were constructed between oat and *A. thaliana*, *T. aestivum*, and *O. sativa* (**Figure 5**). The collinearity analysis revealed 7,418, 56,774, and 94,487 collinearity pairs between oat and *A. thaliana*, *O. sativa*, and *T. aestivum*, respectively. Additionally, three *AsBZR* genes (*AsBZR4*, *AsBZR5*, and *AsBZR6*) demonstrated collinear gene pairs with *AtBEH3* in *A. thaliana* and oat. A total of 13 *AsBZR* genes exhibited syntenic relationships with those in *O. sativa* and *T. aestivum*. The analysis identified 3, 26, and 34 orthologous pairs between oat and *A. thaliana* (**Supplementary Table S5**), *O. sativa* (**Supplementary Table S6**), and *T. aestivum* (**Supplementary Table S7**) respectively. Several *AsBZR* genes showed associated with at least three syntenic pairs in *O. sativa* and *T. aestivum*.

### 3.7 Expression pattern of *AsBZR* genes under osmotic stress

**Figure 6:**
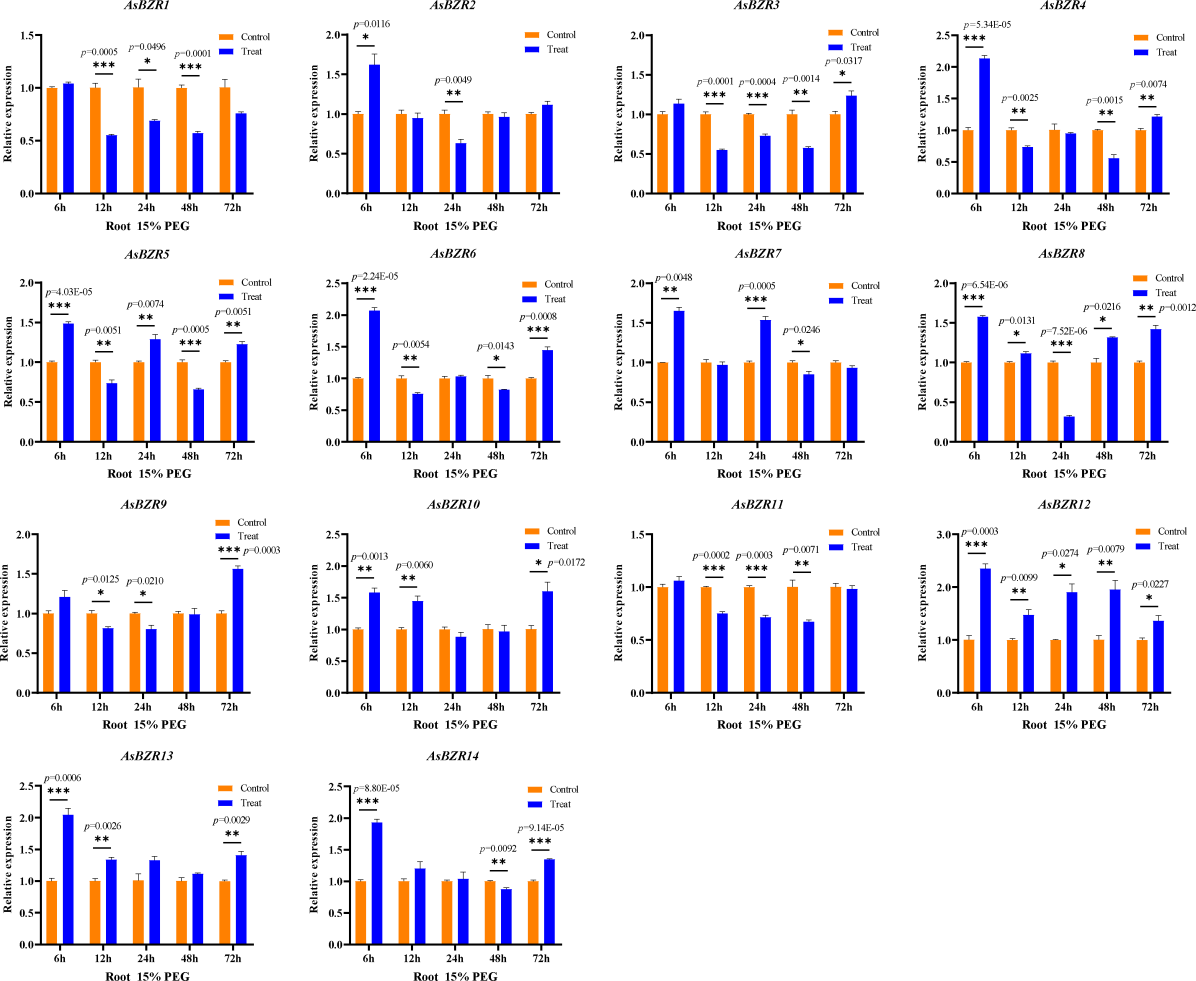
Expression patterns of osmotic stress in roots of *A. sativa* at different time points. The ‘control’ and ‘treat’ groups represent samples of the untreated and treated with 15% PEG 6000, respectively. The *AsActin* gene was used as a control. The error bars indicate the means ± SEMs from three independent experiments. Asterisks indicate significant differences between the ‘treat’ group and the ‘control’ group (*P < 0.05, **P < 0.01, ***P < 0.01, Student’s t-test).

**Figure 7:**
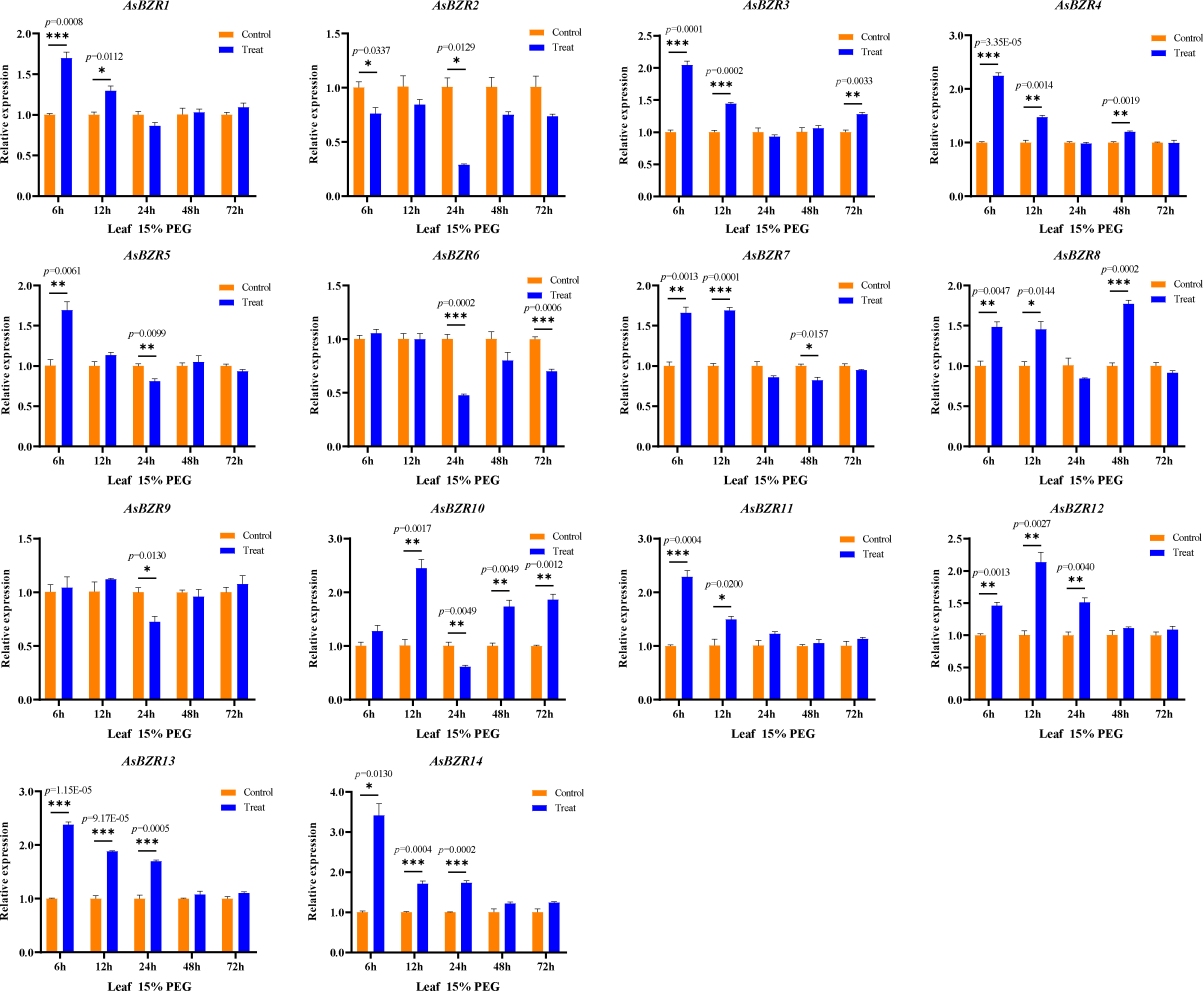
Expression patterns of osmotic stress in leaves of *A. sativa* at different time points. The ‘control’ and ‘treat’ groups represent samples of the untreated and treated with 15% PEG 6000, respectively. The *AsActin* gene was used as a control. The error bars indicate the means ± SEMs from three independent experiments. Asterisks indicate significant differences between the ‘treat’ group and the ‘control’ group (*P < 0.05, **P < 0.01, ***P < 0.01, Student’s t-test).

**Figure 8:**
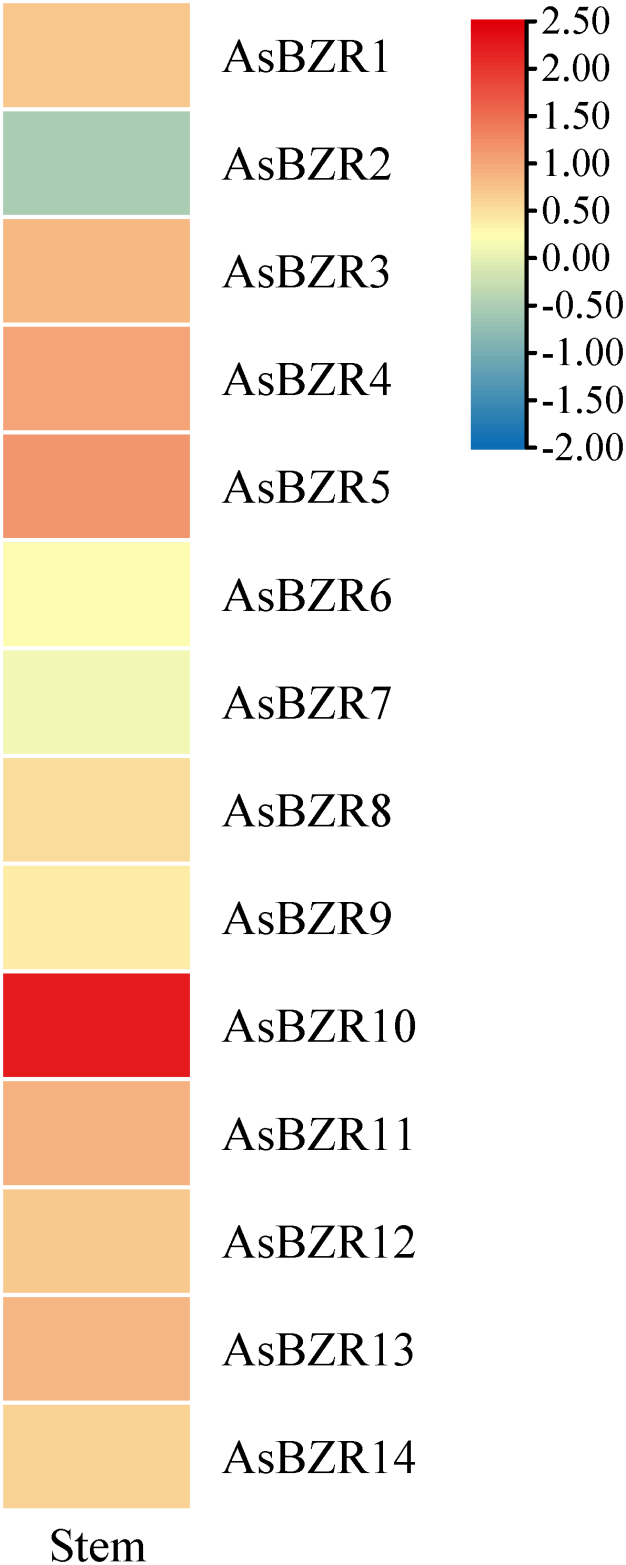
Expression heatmap of osmotic stress in stems of *A. sativa*. Data collected after 24 h of PEG treatment.

To elucidate the response of *AsBZR* genes to osmotic stress, we examined their expression patterns at various time points (6, 12, 24, 48, and 72 h) and in different organs (roots, stems, and leaves) in oat. Relative to the control group, osmotic treatment significantly upregulated *AsBZR10*, *AsBZR12*, and *AsBZR13* in roots, while *AsBZR1* and *AsBZR11* were downregulated. The remaining genes exhibited variable upregulation or downregulation at different time points post-treatment (**Figure 6**). In leaves, *AsBZR1*, *AsBZR3*, *AsBZR4*, *AsBZR8*, *AsBZR11*, *AsBZR12*, *AsBZR13*, and *AsBZR14* were significantly upregulated, while *AsBZR2*, *AsBZR6*, and *AsBZR9* were significantly downregulated (**Figure 7**). At 24 h post-treatment, *AsBZR1*, *AsBZR3*, *AsBZR4*, *AsBZR5*, *AsBZR8*, *AsBZR9*, *AsBZR10*, *AsBZR11*, *AsBZR12*, and *AsBZR14* were upregulated in stems, with *AsBZR2* being downregulated (**Figure 8**). Notably, *AsBZR12* maintained consistent upregulation across all tested organs. The comprehensive expression analysis suggests that *AsBZR12* may play a crucial role in osmotic response.

### 3.8 Subcellular localization of the AsBZR12 protein

**Figure 9:**
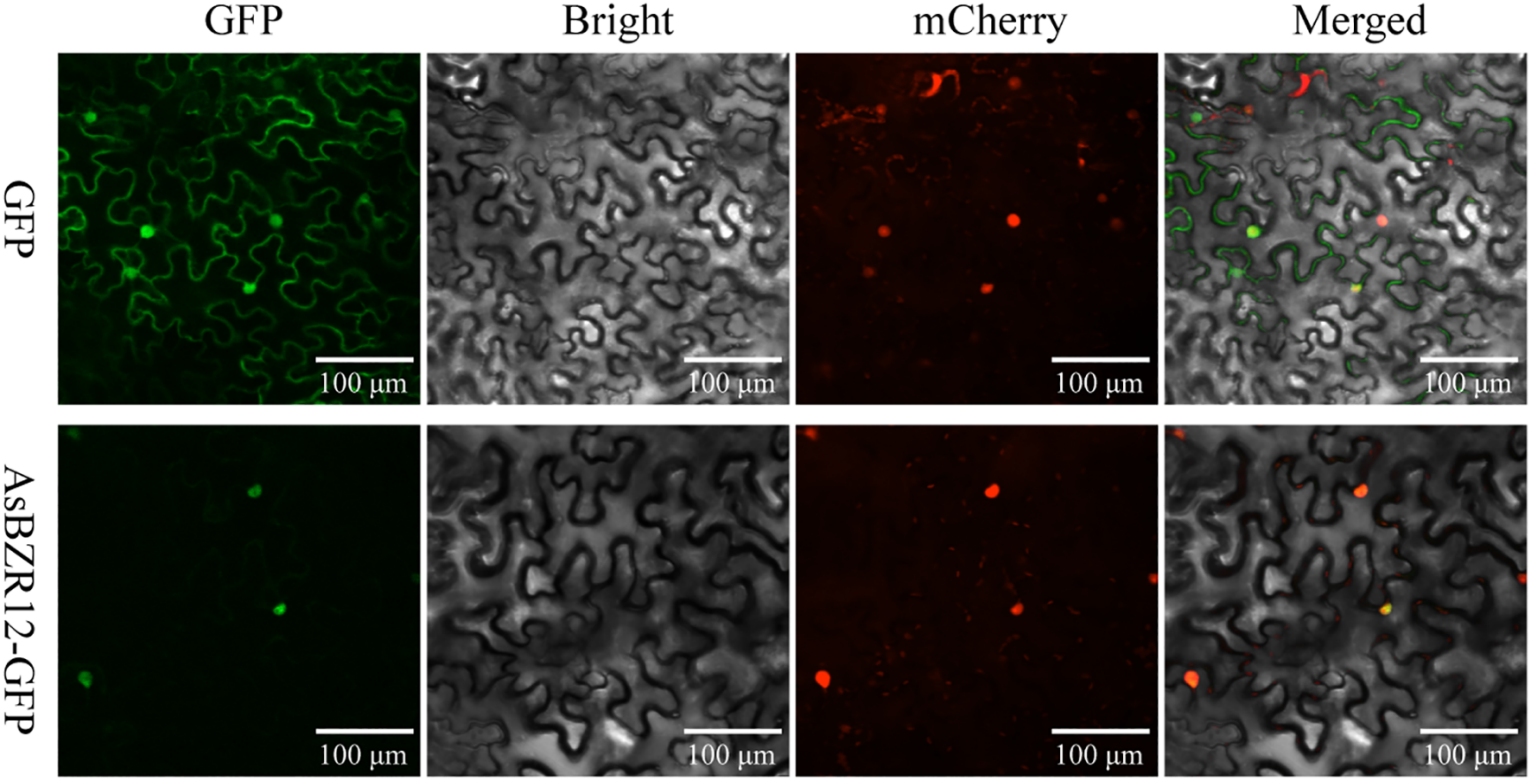
Subcellular localization of AsBZR12. GFP was used as a control. Three independent experiments were performed. Scale bars, 100 μm.

The expression patterns of *AsBZR* genes revealed that *AsBZR12* exhibited consistently high expression levels across all tested organs, suggesting that *AsBZR12* may have a critical function. To investigate the subcellular localization of AsBZR12, we generated an AsBZR12-GFP fusion construct and transiently expressed it in *N. tabacum* leaves. The results revealed that the AsBZR12-GFP fusion protein was localized in the nucleus, confirming our initial prediction (**Figure 9**).

## 4 Discussion

The *BZR* gene family is a key component in the BR signaling pathway, playing an indispensable role in plant growth and development as well as in response to stress. A comprehensive whole-genome analysis of the *BZR* gene family is essential for understanding the functions of BZR proteins in plants. Such analyses have been performed in various species, including *A. thaliana* (Yin et al., 2005), *T. aestivum* (Ullah et al., 2022), *O. sativa* (Ullah et al., 2022), *Zea mays* (Manoli et al., 2018), *S. italica* (Liu et al., 2021) and *Beta vulgaris* (Wang et al., 2019). The number of *BZR* genes identified varies among species: 6 in *A. thaliana*, *O. sativa* and *B. vulgaris*, 16 in *T. aestivum*, 11 in *Z. mays*, and 7 in *S. italica*. The genome size of oat is approximately 11 Gb (He Q. et al., 2024), which is smaller than that of *T. aestivum* but larger than those of *O. sativa*, *Z. mays*, *S. italica*, and *B. vulgaris*. In this study, we identified 14 members of the *BZR* gene family in *A. sativa*, distributed across 12 different chromosomes. The number of members in the *BZR* gene family may be correlated with the size of the plant genome.

To understand the evolutionary relationships and potential functional divergence within the *BZR* gene family in oat, phylogenetic analysis classified the 14 *AsBZR* genes into three groups. This tripartite classification aligns with findings in *C. sativus* (Luo et al., 2023), *B. vulgaris* (Wang et al., 2019), and four dicot species (Wang et al., 2024): *A. thaliana*, *Carica papaya*, *Vitis vinifera*, and *Populus trichocarpa*. Phylogenetic analysis of the AsBZR protein and its orthologs in *A. thaliana*, *O. sativa*, and *T. aestivum* revealed that the *T. aestivum* orthologs within a more closely related branch, indicating a stronger phylogenetic relationship between AsBZR and the *T. aestivum* orthologs. Furthermore, the gene structure and conserved motif identification corroborate the classification results. The widespread presence of motifs 1, 5, 8, and 9 in all *AsBZR* domains emphasizes their significance in maintaining core functions of the *BZR* gene family. Group-specific motifs, such as motif 2 in Group I and motifs 4, 6, and 7 specific in Group III, suggest functional specialization. Additionally, the promoter regions of *AsBZR* genes contain diverse cis-acting elements, including hormone-responsive elements, stress resistance response elements, and tissue-specific expression elements, indicating their critical roles in regulating hormone signaling, responding to stress, and controlling development.

Oats represent a vital food and forage crop in northern China’s arid and semi-arid regions, where drought stress significantly impacts yield and quality (Xu et al., 2024; Zhu S. S. et al., 2024). Studies have shown that drought stress induces the expression of the *BZR* transcription factor in multiple plant species. *BZR* transcription factors play a regulatory role in *Arabidopsis* responses to drought (Ye et al., 2017). Drought stress upregulates *SbBZR1* expression, and its overexpression in *A. thaliana* enhances drought tolerance through activation of antioxidant genes (Li et al., 2025). *ZmBES1/BZR1–4* binds to the downstream target genes *ZmMBP1* and *ZmPum6* to activate their transcription, while simultaneously recruiting the ZmTLP5 protein to form a regulatory complex that further enhances transcriptional activity, thereby negatively regulating drought response (Feng et al., 2025). To elucidate the role of *AsBZR* genes in oat osmotic responses, we analyzed their expression levels under osmotic stress conditions. The expression patterns demonstrate a complex regulatory response to osmotic stress. In roots, most genes exhibit an oscillatory pattern of upregulation, downregulation, and upregulation, except for *AsBZR1*, *AsBZR10*, *AsBZR11*, and *AsBZR12*. In leaves, *AsBZR5*, *AsBZR7*, and *AsBZR10* display a similar oscillatory pattern. Notably, *AsBZR1* and *AsBZR11* demonstrate tissue-specific responses, with downregulation in roots but upregulation in leaves. *AsBZR12* shows significant upregulation across roots, stems, and leaves, with nuclear localization, suggesting its potential role in coordinating osmotic response through regulation of downstream target genes and enhancement of plant osmotic resilience.

We investigated the relationships between *AsBZR12* and its orthologous pairs in wheat and found that *AsBZR12* has three orthologs in wheat, namely *TraesCS7A02G354800* (*TaBZR7-7A*), *TraesCS7B02G272900* (*TaBZR7-7B*), and *TraesCS7D02G368000* (*TaBZR7-7D*). Sequence alignment revealed that these orthologs share a highly conserved BES1_N domain at the N-terminus (**Supplementary Figure S3**). Gene function validation often relies on mutant and transgenic studies. For instance, the *bzr1-1D* mutant has been used to demonstrate that BZR1 inhibits flowering primarily through the suppression of *CO* and *FT* and the activation of *FLC* (Xu et al., 2025). Similarly, mutant and transgenic analyses have confirmed that SlBZR1.7 promotes tomato fruit elongation by positively regulating *SUN* gene expression (Yu et al., 2022). In the present study, we conducted expression analysis but did not perform in-depth functional studies. Future work should focus on creating mutants and conducting transgenic analyses of *AsBZR12* to validate its function.

Gene duplication is a major driving force for gene family expansion and has played a significant role in the evolutionary process of plants (Chen et al., 2024). In wheat, the expansion of the *BZR* gene family is likely mediated by whole-genome duplication (WGD) and segmental duplication events (Kesawat et al., 2021). Oat, like wheat, is hexaploid and have experienced WGD during their evolutionary history. The identification of three pairs of orthologous genes between *AsBZR2*, *AsBZR7*, *AsBZR9*, *AsBZR10*, *AsBZR12*, *AsBZR13* and *TaBZR* suggests that the *BZR* syntenic orthologs may have originated from segmental duplication events. Therefore, we hypothesize that the expansion of the *BZR* gene family in oat is similarly driven by WGD and segmental duplication.

To investigate the genetic relationships and evolutionary dynamics of *BZR* genes across different species, we constructed a collinearity relationship among *A. thaliana*, rice, and wheat. Comparative analysis reveals that more orthologous *BZR* gene pairs are identified between oat and wheat than between oat and either *A. thaliana* or rice. This finding indicates a closer genetic relationship between *AsBZR* and *TaBZR*. To further explore the evolutionary rates of *BZR* genes, we calculated the *Ka/Ks* ratios for *AsBZR* gene pairs and for orthologous *BZR* gene families between oat and rice or wheat. The *Ka/Ks* ratios were consistently less than 1, indicating that *BZR* genes are under purifying selection pressure. Similarly, the *Ka/Ks* ratios of *TaBZR* gene pairs were also less than 1 (Kesawat et al., 2021). These results imply that these genes are highly conserved and likely play crucial roles in maintaining their fundamental functions. Additionally, the *Ka/Ks* ratios between *AsBZR* and *TaBZR* genes are relatively lower than those between *AsBZR* and *OsBZR* genes, further supporting the closer genetic relationship between oat and wheat. This difference may be attributed to the genomic characteristics and evolutionary history of oat, which share many similarities with wheat.

## 5 Conclusion

In this study, we performed a genome-wide identification and characterization of the *BZR* gene family in *A. sativa*, identifying 14 *AsBZR* genes divided into three groups. The promoters of all *AsBZR* genes contain enriched elements response to phytohormone and environmental stress, demonstrating strong purifying selection pressure throughout evolution. Through qRT-PCR analysis, *AsBZR12* demonstrated significant involvement in osmotic stress response, with AsBZR12 showing nuclear localization. These findings elucidate the sequence characteristics of the *AsBZR* gene family and establish a foundation for future investigations into the osmotic stress resistance functions of *AsBZR* genes in oat.

## Data Availability

The original contributions presented in the study are included in the article/**Supplementary Material**. Further inquiries can be directed to the corresponding author.
