# Supplementary material for: Genome-wide identification and characterization of the Brassinazole-resistant gene family and associated responses to osmotic stress in Avena sativa
Source: Front Plant Sci. 2025 Aug 14;16:1616026. doi: 10.3389/fpls.2025.1616026 (PMC12391064; doi:10.3389/fpls.2025.1616026)
Supplement: Supplementary file 2 [file Image1.pdf]

Supplementary Material

Supplementary Figures

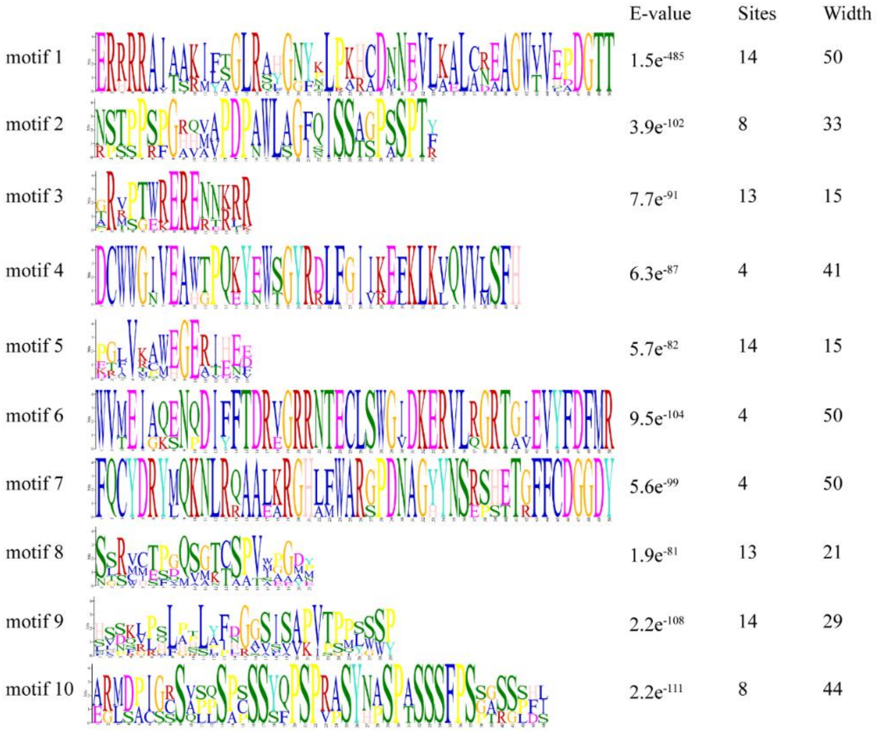

Supplementary Figure 1. The detailed information of conserved motifs in AsBZR proteins.

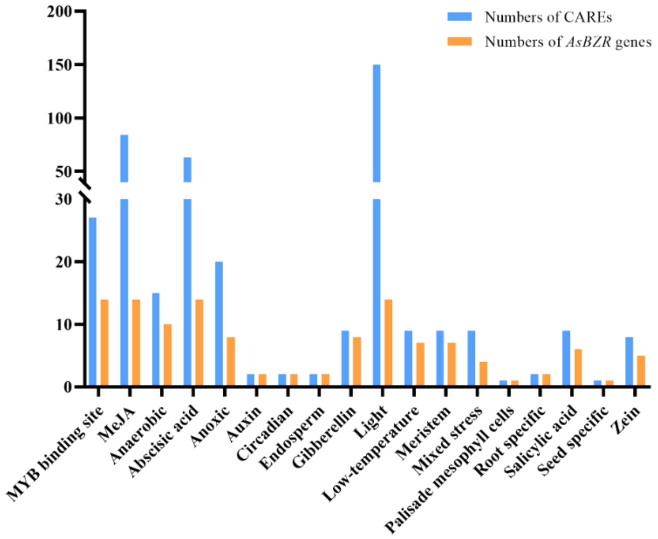

Supplementary Figure 2. Number of each cis-acting element in the promoter region of *AsBZR* genes.

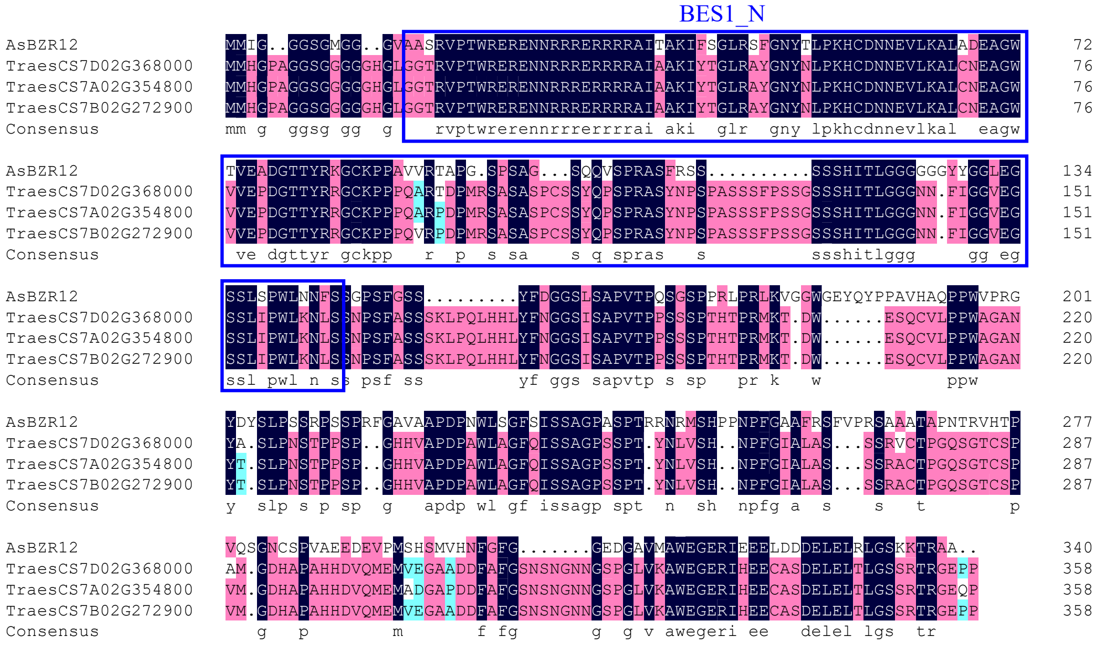

Supplementary Figure 3. Amino acid sequence alignment of AsBZR12 and its orthologous gene in wheat. The blue frame indicated the BES1_N domain.
